# Client experience of food assistance programs among adults in the United States: a qualitative evidence synthesis protocol

**DOI:** 10.3389/fpubh.2023.1193451

**Published:** 2023-08-31

**Authors:** Elizabeth C. Rhodes, Kate Nyhan, Ngozi Okoli, Kathleen O’Connor Duffany, Maria Elena Rodriguez, Benjamin Perkins, Daniel Ross, Rafael Pérez-Escamilla

**Affiliations:** ^1^Hubert Department of Global Health, Rollins School of Public Health, Emory University, Atlanta, GA, United States; ^2^Harvey Cushing/John Hay Whitney Medical Library, Yale University, New Haven, CT, United States; ^3^Department of Environmental Health Sciences, Yale University School of Public Health, New Haven, CT, United States; ^4^Department of Social and Behavioral Sciences, School of Public Health, Yale University, New Haven, CT, United States; ^5^DAISA Enterprises, LLC, South Hadley, MA, United States; ^6^Wholesome Wave, Bridgeport, CT, United States

**Keywords:** person-centered, food assistance program, qualitative evidence synthesis, systematic review, United States

## Abstract

**Introduction:**

Policymakers, health practitioners, and other key partners are increasingly focused on ensuring that clients of food assistance programs have positive experiences, a key aspect of high-quality programming. The objectives of this review are to describe the experiences of clients participating in food assistance programs in the United States (US) and to identify ways that these programs promote or hinder positive experiences.

**Methods and analysis:**

We will conduct a qualitative evidence synthesis with partners from food security organizations and community members. Peer-reviewed literature will be systematically searched in Scopus, CINAHL, and AGRICOLA. To identify grey literature, we will use Google’s programmable search engine. This review will consider sources that present results of primary qualitative studies that focus on at least one food assistance program in the US and explore the perspectives of adult clients. Only sources published in English or Spanish from 2007 onward will be included. Multiple reviewers will screen articles for inclusion and extract data from articles that meet the inclusion criteria, using a structured data extraction tool. Thematic synthesis or meta-ethnography may be appropriate approaches for synthesizing the extracted data. The final selection of synthesis method will be determined once the set of primary qualitative studies to be included in the review is complete and the type of data presented in these studies is known. We will assess the methodological quality of the included studies using the CASP (Critical Appraisal Skills Programme) tool for qualitative studies and assess the confidence in the review findings using the GRADE-CERQual (Confidence in Evidence from Reviews of Qualitative research) approach.

**Discussion:**

The findings of this review will inform the development of measures to assess client experience and quality improvement efforts.

## Introduction

In 2020, one in ten households in the United States (US) were food insecure (1). To alleviate food insecurity, a variety of food assistance programs have been implemented to increase access to safe and nutritious food for millions of individuals and families each year. For example, the US Department of Agriculture (USDA) Supplemental Nutrition Assistance Program (SNAP) serves approximately 43 million adults with low incomes annually (2). In 2021, the Special Supplemental Nutrition Program for Women, Infants, and Children (WIC) served about 6.2 million participants each month (3). Produce prescription programs are increasingly integrated into healthcare systems and provide vouchers or debit cards for free or discounted fruits and vegetables to patients experiencing food insecurity (4). People who are or at-risk for experiencing food insecurity use food pantries, food banks, and other community-based food assistance programs. Evidence shows that many food assistance programs have a positive impact on a wide range of metrics assessing food behaviors (e.g., fruit and vegetable intake, dietary quality), food security, health (e.g., disease self-management, mental well-being), and healthcare utilization (e.g., emergency department visits) and cost (4, 5). Expanding effective food assistance programs is a major focus among policymakers, funders, and implementers, especially during times of economic crisis or public health emergencies like the COVID-19 pandemic (6, 7). Simultaneously, there is widespread recognition that it is important to promote positive experience of food assistance programs among individuals participating in these programs, hereafter referred to as clients (6, 8).

Positive client experience is a key indicator of person-centeredness, one of the six dimensions of high-quality health services identified by the National Academies of Sciences, Engineering, and Medicine (9). A person-centered service is “respectful of and responsive to individual preferences, needs, and values” (9). Positive client experience of food assistance programs means that clients have good interpersonal interactions with people delivering the program, such as being treated with respect and dignity. The design and delivery of programs can also be person-centered to promote positive client experiences. For example, programs can be designed and delivered in a way that ensures wait times are short, services are easy to navigate, and services are reflective of clients’ preferences, needs, and values (10).

Previous studies have explored client experiences of different types of food assistance programs. In a qualitative study that explored clients, experiences of a produce prescription program, Schlosser and colleagues found that clients felt program providers cared about them because they took time to discuss eating patterns and nutrition goal-setting (11). Qualitative studies have also described how the design of food assistance programs can influence client experience. Limited hours of food banks and food pantries, combined with significant time spent waiting in long lines, have been found to contribute to negative client experience, particularly since many people who experience food insecurity have jobs with inflexible schedules or are unable to take time off work (12, 13). Bruckner and colleagues found that clients of food banks and food pantries described “red tape,” such as long application forms and regulations requiring proof of financial hardship, as negative experiences because it made them feel judged by staff and created feelings of stress, anxiety, and shame (12). There is accumulating evidence that the re-design of government food assistance programs, such as converting from paper vouchers to electronic benefits transfer (EBT) cards that work like debit cards, have improved client experience. For example, Zimmer and colleagues conducted in-depth interviews with WIC participants and found that food shopping via EBT cards compared with paper vouchers improved participants’ experiences, since they were able to have a faster and more discrete checkout (14).

Positive client experience of food assistance programs is important for multiple reasons. First, positive client experience is a right; all individuals have the right to be treated with respect and dignity (15). Second, positive client experience may promote the use of food assistance programs. Gundersen argued that the main reason SNAP has achieved a high participation rate is that the program treats recipients with dignity and autonomy – for example, by allowing SNAP recipients to shop in the over 260,000 stores that accept SNAP benefits so that they can shop in traditional retail stores like their neighbors rather than limiting options for food shopping, and by giving recipients the ability to make food purchasing decisions that meet the needs and preferences of household members instead of restricting foods that recipients can buy with their benefits (16). On the other hand, a well-documented barrier to seeking food assistance is stigma and shame felt during the process of accessing food assistance due to a lack of privacy and visibility when using program services, as well as judgment from staff enrolling individuals in programs or cashiers at grocery stores (13, 17). When food offered by food pantries is not the type desired or of poor quality, clients view this as a sign of disrespect from program providers and opt to not take the food (18). Additional barriers to accessing food assistance include challenges in locating programs, lack of transportation to pick up food, and inconvenient program hours of operation (17, 19–21).

Although there is a sizeable body of literature describing clients, experiences of food assistance programs, there has not yet been an effort to systematically synthesize existing research to describe what is known about client experience. The objectives of this systematic review are to describe the experiences of clients participating in food assistance programs and to identify ways that these programs promote or hinder positive client experience. Of note is that a preliminary search of Google Scholar and PubMed was conducted and no current or in-progress reviews on the topic were identified.

## Methods and analysis

### Review questions

1. What are clients, experiences of food assistance programs among adult clients in the US?

2a. What practices promote positive client experience of food assistance programs among adult clients in the US?

2b. What practices hinder positive client experience of food assistance programs among adult clients in the US?

### Eligibility criteria

#### Population

This review will consider articles that focus on adult clients of food assistance programs in the US.

#### Concept

The concept of interest is client experience of food assistance programs in the US. The USDA characterizes food insecurity as a lack of continuous access through socially acceptable means to nutritious and safe foods in the amounts needed for a healthy and active life (22). The USDA also acknowledges that respecting the cultural preferences of people for diverse foods is a key component of the food security construct (22). For the purpose of this review, any program that is designed to address food insecurity will be considered a food assistance program.

#### Context

Since food assistance programs can be delivered in a variety of settings, this review will not include specific service settings as inclusion or exclusion criteria. We will consider any service setting in the US, including but not limited to healthcare facilities, community organizations, and government programs.

#### Types of sources

This review will consider primary qualitative studies in both the published literature and grey literature.

### Co-creation model

Knowledge users will be involved in all stages of the review. Knowledge users include leaders and staff members from Wholesome Wave and DAISA Enterprises (DAISA), organizations addressing disparities in diet-related diseases by making fruits and vegetables more accessible and affordable to community members with low incomes through systems changes. These organizations are also leading efforts to make food assistance programs more person-centered and equitable. Authors from the Yale School of Public Health (YSPH) and Emory University have worked alongside Wholesome Wave and DAISA leaders and staff members to develop this protocol. Wholesome Wave and DAISA leaders and staff approved the review questions and plan for data synthesis and presentation and contributed to the development of the search strategy. They will be engaged in the review process – for example, by offering suggestions on relevant grey literature sources and providing input on the synthesis and presentation of results. They will also disseminate the review findings through their networks of community-based and advocacy organizations. The Yale-Griffin Prevention Research Center Community Advisory Group, comprised of representatives from community agencies and organizations and community members, will also be engaged in the review process. This work will build on an established partnership between the authors and Wholesome Wave and DAISA leaders and staff, as well as a strong collaboration with the Yale-Griffin Prevention Research Center Community Advisory Group. Using a co-creation model will increase the relevance of the research for health practitioners and policymakers working to improve the quality of food assistance programs (23, 24). An additional benefit of co-creation of reviews is that it will improve the use of results by Wholesome Wave and DAISA leaders and staff, as well as the dissemination of findings to other relevant partners (23).

### Search strategy

The search strategy was developed in collaboration with a research librarian (KN) and peer reviewed by a medical librarian. The published literature will be systematically searched in Scopus, CINAHL, and AGRICOLA using controlled vocabulary and free-text terms/keywords combining two concepts: (a) food assistance programs; and (b) client experience (see Table 1). We developed the search strategy by reviewing the search strategies in published reviews on adjacent topics (for example, a scoping review of food prescription programs (25)) and drafting a concept table of potential search terms that were refined with input from subject matter experts and practitioners based at Wholesome Wave and DAISA. After an initial search in Scopus yielded a large number of results set with low specificity, we revised the queries iteratively, spot-checking the “marginal” results each time we considered removing an OR statement from the search. For example, we removed the abbreviation “SNAP” from the query for food assistance programs after confirming through testing that authors who use the abbreviation also write out the full name of the program or use the phrase “food assistance,” in almost all cases. The search strategy was then validated by reviewing the search results to determine whether 11 articles that were known to the authors and that met the inclusion criteria were retrieved through the search. During this validation process, we found that one article was not retrieved by our query for articles which use qualitative methods, or the qualitative hedge. We therefore added additional terms to our qualitative hedge, and then successfully retrieved the last validation article.

**Table 1:** Search strategy.

(TITLE-ABS-KEY (“food assistance” OR “nutritional assistance” OR “supplemental nutrition assistance program*” OR snap-ed OR wic OR “women infants and children” OR “expanded food and nutrition education program*” OR efnep OR “child and adult care food program*” OR cacfp OR “food distribution program on indian reservations” OR fdpir OR “commodity supplemental food program*” OR csfp OR “wholesome wave” OR “food stamp*” OR “food buck*” OR “food bank*” OR foodbank* OR “food pantr*” OR “school pantr*” OR “community pantr*” OR “emergency food system*” OR “emergency food operation*” OR “emergency food” OR “soup kitchen*” OR “food charit*” OR “food as medicine” OR “food is medicine” OR ((food OR produce OR “fruit and vegetable”) W/2 (prescribing OR prescription* OR voucher* OR rx OR incentive* OR pharmacy OR pharmacies)) OR “fruit and vegetable incentive*” OR “nutrition incentive program*” OR “farmer* market*” OR “mobile market*” OR farmacy OR farmacies OR “food referral*” OR “social prescribing” OR “social prescription*” OR ((food OR vegetable) W/1 (box* OR hamper*)) OR “emergency food assistance program*” OR tefap OR “older americans act nutrition program*” OR “home-delivered nutrition program*” OR “congregate nutrition program*” OR “meals on wheels” OR “senior farmer* market* nutrition program*” OR sfmnp OR ((pantr* OR aid OR assistance) AND (hunger OR “food insecur*” OR “food secur*” OR “farming for life”)) AND (TITLE-ABS-KEY (satisfaction OR satisfied OR “patient* value*” OR usability OR ux OR “service design” OR qualitative OR ethnograph* OR interview* OR “focus group*” OR “mixed methods” OR open-ended OR ((patient* OR user* OR consumer* OR client* OR individual OR people*) W/1 (perspective* OR opinion* OR view* OR experience OR experiences OR voice)))

To identify grey literature, we will use Google’s programmable search engine, which allows for searching grey literature across specified websites. To determine which websites to specify in the search, we will work with partners from Wholesome Wave, DAISA, and the Yale-Griffin Prevention Research Center’s network of partners to compile a list of government and non-governmental websites that may include relevant grey literature.

To identify additional articles, backward citation chaining will be carried out by reviewing reference lists of included articles and forward citation chaining will be conducted using citationchaser (26). Additionally, experts with research or practice-based experience in the health and food security fields will be contacted. These experts may include researchers at academic institutions, program managers at community-based organizations, health practitioners at government agencies, and others. We will restrict the search to articles published in the past 16 years (2007–2023). The rationale for this date restriction is that food assistance programs, such as SNAP and WIC, underwent significant changes as a result of the 2008 Farm Bill (27) or substantial program benefits changes (28), and we are interested in client experience of these programs in their current and most recent form. Records will be kept of all searches and search results.

Following standards for reporting evidence synthesis, we will report relevant items from PRISMA 2020, PRISMA-S, and ENTREQ (29–31).

### Study selection

All citations and abstracts of articles identified by the search will be uploaded into Covidence (Veritas Health Innovation, Melbourne, Australia) and duplicates will be removed. Next, the titles and abstracts of articles will be screened in Covidence for inclusion by multiple reviewers using standardized inclusion criteria: (a) presents results of a primary qualitative study; (b) focuses on at least one food assistance program to promote food security; (c) focuses on at least one food assistance program in the US; (d) discusses client experience from the perspectives of clients; and (e) is published in English or Spanish. As indicated above, no exclusions will be made due to the setting in which the food assistance program is delivered, since food assistance programs are delivered in a variety of settings. Studies that focus solely on client experience among children or adolescents will be excluded.

Two reviewers will be trained to apply the inclusion and exclusion criteria. To ensure consistency, reviewers will screen the same 200 titles and abstracts in Covidence. Inter-rater reliability (IRR), measured as percent agreement, will be assessed, and disagreements will be identified and resolved through discussion with reviewers. If the IRR and the reviewers’ contributions to the conflict resolution discussion suggest that there are different understandings of the inclusion and exclusion criteria, additional training will be provided to address those differences. Reviewers may also screen an additional set of the same 200 titles and abstracts, which would allow for IRR to be assessed and any disagreements to be identified and resolved through further discussion. Once reviewers reach alignment, each reviewer will screen articles independently in Covidence. After the abstract screening phase, the full text of each remaining article will be retrieved and imported into Covidence. Two reviewers will screen the full text of one-fourth of the articles. IRR will be assessed, and disagreements will be identified and resolved through discussion with reviewers prior to them screening the full text of articles independently.

During the searching, screening, and selection process, new potentially relevant terms or concepts may come to light. As such, we may modify and expand the proposed search during the review process (32).

### Data extraction

Data will be extracted by multiple reviewers using a data extraction tool (see Table 2). We will pilot the draft data extraction tool on a subset of articles included in the review to assess its feasibility and refine the tool as needed. Modifications to the extraction tool will be detailed in the full review. To ensure consistency of data extraction, data will be extracted from one-third of the included articles by at least two reviewers and then compared. Any differences that arise between the reviewers will be identified and resolved through discussion. Throughout the data extraction process, quality checks of extracted data will be conducted.

**Table 2:** Preliminary data extraction tool.

Basic article information	DOI
	Authors
	Year
	Journal
	Funding support
	Open access (yes/no)
Characteristics of food assistance program	Type (e.g., food bank, produce prescription program, SNAP)
	Geographic location
	Target population(s)
	Organization(s) delivering the program
Study objectives	Objective(s)
Qualitative methods	Study design
	Study setting
	Sample
	Recruitment methods
	Data collection method(s)
	Data analysis method(s)
Qualitative findings	Client experiences
	Practices that promote positive and/or negative client experience
	Participant quotations

### Data synthesis and presentation

Qualitative data may be synthesized using thematic synthesis or meta-ethnography, which are both appropriate approaches that have been used to understand experiences of interventions among those who receive them (24). A thematic synthesis has flexibility in that it allows for the incorporation of qualitative data that are “thin” and/or “thick” in the generation of themes, whereas meta-ethnography requires included studies to predominately have “thick” data (24). The final selection of a qualitative evidence synthesis approach will therefore be determined once the set of primary qualitative studies to be included in the review is complete and the type of data presented in these studies is known (24). To inform appropriate selection, the RETREAT guidance for choosing an approach for a qualitative evidence synthesis will also be considered (33). Review findings will be presented in a diagram and/or table that aligns with the objectives of the review.

### Assessment of methodological limitations and confidence in review findings

To assess the methodological limitations of each study contributing the review findings, the Critical Appraisal Skills Programme (CASP) tool will be used (24). A GRADE-CERQual approach will be used to assess how much confidence to place in the individual findings from our qualitative evidence synthesis. The level of confidence in the evidence refers to the extent to which a review finding is a reasonable representation of the phenomenon of interest (34). In this approach, the assessment of confidence for individual review findings is based on four components: (1) the methodological limitations of the qualitative studies contributing to a review finding, (2) the coherence of the review finding, (3) the adequacy of data supporting a review finding and (4) the relevance of the data from the primary studies contributing to a review finding to the review question (34). The assessments of these components will be used to produce an overall assessment of confidence (high, moderate, low, or very low) in each review finding (34). The investigators who will conduct these assessments have the required skills to apply the CASP tool and GRADE-CERQual approach, including an understanding of systematic review methodology and an understanding of the principles of qualitative research.

## Ethics and dissemination

Since this review will not involve human subjects, it was not necessary for this protocol to be reviewed and approved by an Institutional Review Board. The results of the review will be presented at scientific meetings and in a peer-reviewed journal article. Through presentations and research briefs, the findings will be shared with community-based organizations, clinics and hospitals, decision makers, and other relevant partners delivering and funding food assistance programs.

## Discussion

This systematic review will synthesize qualitative findings on the experiences of clients of food assistance programs in the US. The review protocol was developed by researchers, partners, and research and medical librarians in response to a need we identified in the food assistance field based on our knowledge of the literature and the information needs of programmatic partners and government agencies that are increasingly focused on promoting positive client experiences (35).

A major strength of the review is that it will apply a co-creation model in which knowledge users will be involved throughout the review process, which will increase the relevance of the review findings for practice and policy efforts to promote positive client experience of food assistance programs (23, 24). Another strength is that the review will include literature focused on different types of food assistance programs. The findings of our qualitative evidence synthesis may yield an in-depth understanding of client experiences across a range of programs and illuminate ways in which diverse programs promote or hinder positive experiences. Finally, the GRADE-CERQual approach offers a transparent method for assessing overall confidence in the findings from qualitative evidence syntheses (36). Furthermore, this approach may support the use of qualitative evidence in decision making for health and social interventions like food assistance programs (36).

This review also has limitations that are important to note. One limitation is that grey literature – while important for a complete picture of knowledge on this topic – is impossible to search in an exhaustive and transparent fashion. Our plan to create a Google programmable search engine (formerly known as a custom search engine) brings a level of reproducibility to the process, but the process of identifying and screening relevant grey literature remains less transparent, compared with scholarly literature indexed in bibliographic databases. An additional limitation is that the three information sources we are using are all paywalled,^1^ and as a result, while our detailed search strategies are available for any interested reader, only people who are affiliated with academic institutions will be able to easily re-run our searches.

This review is the first step in a larger project to make produce prescription programs more person-centered and equitable. The findings will be used to inform the development of a measure to assess client experience of produce prescription programs. Based on the findings, we will also be able to propose approaches that produce prescription programs can use to promote positive and prevent negative client experiences. Future projects, grounded in evidence from this review, could be undertaken at the national, state, and local levels to improve client experience of other types of food assistance programs and make progress toward the vision of high-quality food assistance. This review may also identify gaps in the literature on client experience of food assistance programs. In doing so, it can inform the development of a future research agenda to generate the knowledge needed to guide quality improvement and assurance systems for the quite diverse food assistance programs in the US, taking respect, dignity, and equity considerations into account. The Biden-Harris Administration National Strategy on Hunger, Nutrition, and Health released in 2022 put forward a goal of strongly linking food and nutrition security with health security with the specific goal of providing support to low-income families to increase healthy eating by 2030 (37). As shown by the COVID-19 pandemic, food availability, accessibility, and affordability can rapidly deteriorate, increasing the prevalence and worsening the severity of food insecurity (19, 35). Food assistance programs have a key role to play in protecting individuals and families against the repercussions of public health emergencies by mitigating their impacts on food security (35). As efforts to strengthen and expand food assistance programs intensify, a strong focus on person-centered programming is imperative.

In conclusion, the findings of this review can contribute to a better understanding of clients’ experiences with food assistance programs which is needed to, among other things, develop person-centered metrics of clients’ experiences and satisfaction, understand how these experiences mediate or modify the impacts of programs, and develop quality assurance programs to promote positive client experiences at all touch points. Additionally, the findings will bring to the forefront the voices of people participating in food assistance programs – a crucial step in improving the co-design, implementation, evaluation, and sustainability of food assistance programs.

## Author contributions

ER, RP-E, and KO'C conceptualized the systematic review. ER, KN, and NO developed the protocol with input from all co-authors. All authors critically reviewed and approved the submitted manuscript.

## Funding

The development of this protocol was made possible through the support of the Yale Griffin Prevention Research Center Cooperative Agreement Number 5 U48DP006380-02-00 funded by the US Centers for Disease Control and Prevention, Prevention Research Center Program (PI: RP-E). Its contents are solely the responsibility of the authors and do not necessarily represent the official views of the US Centers for Disease Control and Prevention or the US Department of Health and Human Services. The development of this protocol was made possible by the Walmart Foundation. The protocol was developed by the authors alone and do not necessarily reflect the opinions of the Walmart Foundation.

## Conflict of interest

MR and DR are employed by DAISA Enterprises, LLC.

The remaining authors declare that the research was conducted in the absence of any commercial or financial relationships that could be construed as a potential conflict of interest.

## Publisher’s note

All claims expressed in this article are solely those of the authors and do not necessarily represent those of their affiliated organizations, or those of the publisher, the editors and the reviewers. Any product that may be evaluated in this article, or claim that may be made by its manufacturer, is not guaranteed or endorsed by the publisher.

## Footnotes

^1^The database AGRICOLA is available to everyone on the internet via the new SEARCH discovery layer of the National Agriculture Library. However, some features that we used (including adjacency operators and exploded subject headings) are available only when accessing the database AGRICOLA through platforms provided by paid vendors, such as Ovid.
